# Service Reliability Test Method for Anticorrosion Coatings on the Compressor Outlet Pipelines of Natural Gas Stations

**DOI:** 10.1021/acsomega.2c06648

**Published:** 2023-02-10

**Authors:** Hailiang Nie, Weifeng Ma, Kang Deng, Guangqiang Zhao, Ying Mao, Junjie Ren, Wei Dang, Ke Wang, Jun Cao, Tian Yao, Xiaobin Liang

**Affiliations:** † Institute of Safety Assessment and Integrity, State Key Laboratory for Performance and Structure Safety of Petroleum Tubular Goods and Equipment Materials, Tubular Goods Research Center of CNPC, Xi’an 710077, China; ‡ No. 10 Oil Production Plant of PCOC, Jiayuguan 745000, Gansu, China; § PetroChina Coalbed Methane Company Limited, Beijing 100028, China; ∥ Southwest Gas Production Plant of PetroChina Zhejiang Oilfield Company, Yibin 645250, Sichuan, China

## Abstract

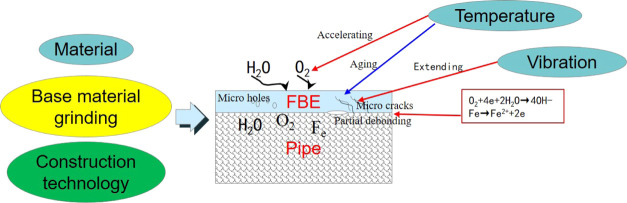

Compressor outlets are subject to high temperatures and vibrations; when pipelines are subject to such conditions, degradation of the anticorrosive layer on the pipeline is likely. Fusion-bonded epoxy (FBE) powder coating is the most common type of anticorrosion coatings on compressor outlet pipelines. It is necessary to study the reliability of anticorrosive layers in compressor outlet pipelines. In this paper, a service reliability test method for the corrosion-resistant coatings of compressor outlet pipelines of natural gas stations is proposed. Testing involving the simultaneous exposure of the pipeline to high temperatures and vibrations is conducted to evaluate, on a compressed timescale, the applicability and service reliability of FBE coatings. The failure mechanism of FBE coatings exposed to high temperatures and vibrations is analyzed. It is found that, due to the influence of initial imperfections in the coatings, FBE anticorrosion coatings typically do not meet the standard requirements for use in compressor outlet pipelines. After simultaneous exposure to high temperatures and vibrations, the impact resistance, abrasion resistance, and bending resistance of the coatings are found not to meet the requirements for their intended applications. It is therefore suggested that FBE anticorrosion coatings be used with extreme caution in compressor outlet pipelines.

## 1 Introduction

In recent years, more and more attention has been paid to the corrosion protection layer damage of compressor outlet pipeline in oil and gas station. According to the investigation, the compressor outlet is in the state of high-frequency vibration, and the temperature of pipeline can reach up to 70 °C.^1,2^ At present, the index requirements about high temperature and vibration of anticorrosive layer have not been included in the relevant standards,^3−5^ so it is urgent to study the applicability and service reliability of anticorrosive layer products in the compressor export so as to provide a basis for the selection of anticorrosive layer.

To study the damage mechanism of anticorrosive coating, numerous research methods have been proposed. The most commonly used method is to study the influence of different processes on the properties of anticorrosion coatings through combinations of micro and macro tests.^6^ Different types of anticorrosive coatings have been studied to establish their resistance to degradation as a result of exposure to high temperatures; the construction of these coatings can then be optimized to improve their high-temperature stability.^7^ Considerable research has been undertaken to study the failure process of anticorrosion coatings and the effect of this process on pipelines by creating defects in the corrosion coating.^8^

There exists extensive literature on the mechanisms that cause damage to anticorrosive coatings. For some coatings that are stable at high temperatures, it has been found that the high-temperature stability of the coating is due to a particular microstructure or the tribological and oxidation characteristics of the coating when it is exposed to high temperatures.^9−11^ The influence of a given component in the coating on the properties of the coating, such as high-temperature resistance and oxidation resistance, has also been studied.^12,13^ It is hypothesized that porosity is an important factor in determining the resistance of a coating to cathode stripping.^14^ The permeability of a coating to ions, water, and oxygen depends on its porosity; materials with high porosities typically also exhibit high permeabilities, which means that water and oxygen are more likely to seep through the coating to the base material. The mechanism behind the adhesion of fusion-bonded epoxy (FBE) coatings has also been studied;^15^ such studies aim to identify and quantify the primary factors determining the adhesion of such coatings to the base materials. Molecular dynamics simulations have been used to study the effect of temperature, humidity, internal stress, and other variables on the degradation rate in the stratification process. Impregnation tests have been undertaken on FBE monolayer coatings at various temperatures to determine the rate constant required for the calculation of activator. The working mechanism behind anticorrosive coatings has been hypothesized,^16^ and a series of relatively simple tests that measure the properties determining the effectiveness of the coating in a quantitative manner have also been suggested. Such investigations permit the development of superior coatings and provide guidance on appropriate test methods to determine the properties of a given coating.

Considerable research has been devoted to the study of the dependence of the failure model of an anticorrosive layer on various parameters from different perspectives. Ge^17^ described the effect of temperature on the sliding wear characteristics of AlNiTi amorphous coatings. Xuan^18^ experimentally investigated the effect of temperature profiles, microstructural evolution, and wear resistance of plasma-sprayed NiCrBSi coatings under different powers in a vertical remelting way. Zhao^19^ investigated the corrosion behavior and cracking susceptibility of disbonded coatings of X80 steel pipelines via both numerical and experimental methods. Tchoquessi Diodjo^20^ investigated the characteristics of the stress evolution in multilayer polymer coatings applied to the pipeline structures subject to thermal and pressure loads. Wu^21^ described the effect of coating temperature and atmosphere on the performance of scheelite coatings on SiC fibers.

The existing literature has established the damage mechanisms of FBE anticorrosion coatings subject to a selection of working conditions and provided a theoretical basis for the corrosion protection of pipelines. No work has been devoted to the damage mechanism of FBE anticorrosion coatings in buried pipelines when the pipeline is subject to the combined effect of high temperature and vibrations.

In this paper, a service reliability test method is proposed that is applicable to the corrosion-resistant coating of compressor outlet pipelines in natural gas stations. The developed experimental system, which exposes samples to high temperatures and vibrations, is used to simulate the service conditions of compressor outlets. The FBE anticorrosive coating on the compressor outlet of a natural gas station was used to test the performance and reliability of the coatings. The micro failure mechanism of an FBE coating that was simultaneously exposed to high temperatures and vibrations was established.

## 2 Methods

Li established that when water infiltrates the anticorrosion layer, it induces swelling and dissolution or even chemical decomposition; this leads to the water penetrating to the steel surface, which results in the stripping of the protective layer from the outer surface of the pipeline and initiates corrosion. Therefore, soil and soil simulation solutions were used in the experimental work presented here to ensure a comprehensive characterization of the performance of the anticorrosion layer.

**Figure 1:**
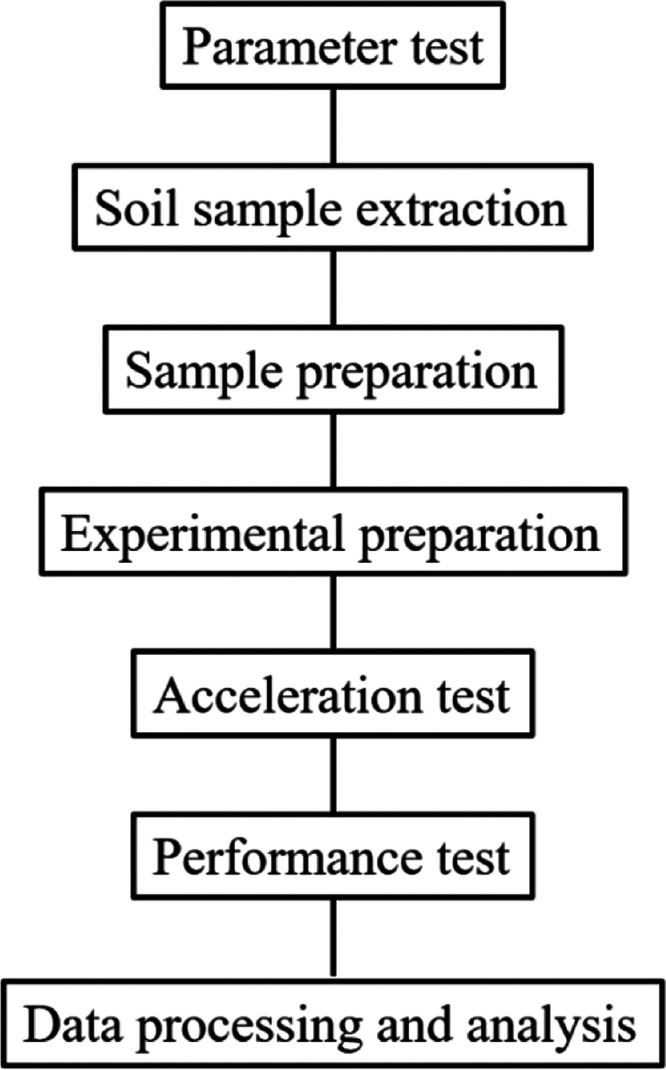
Processes within the reliability testing procedure for anticorrosion coatings on compressor outlet pipelines.

Based on the insight provided by the above theoretical work, a service reliability test method for the compressor outlet pipeline of natural gas stations was developed that includes the following processes (as shown in Figure 1): parameter testing, soil sample extraction, sample preparation, experimental preparation, accelerated testing, performance testing, and data processing and analysis.

Here, we describe each step of the reliability testing procedure depicted in Figure 1.

### 2.1 Step 1: Parameter Testing

Parameter testing includes a vibration spectrum test and temperature test of the compressor outlet pipeline. The region of the compressor outlet pipeline where it is most likely that the anticorrosion coating fails should be selected for testing; in steps 1–3, the same station should be considered.

Conventional contact or noncontact temperature testing equipment can be used for the temperature test. A minimum of three measurements should be taken at the same location at different times; a time period of more than 3 h should be left between each measurement, and at a given longitudinal position, a minimum of four measurements at different points on the circumference of the pipeline should be considered.

Conventional vibration spectrum testing equipment can be used for the pipeline vibration spectrum test. At a given longitudinal point, four positions on the circumference of the outlet pipe should be tested, and the vibration spectrum along the axial and the vertical directions of the pipeline should be tested at each position on the circumference of the pipeline. The vibration spectrum at a given position on the circumference of the structure should be continuously tested to establish the entire characteristic spectrum of the vibration is captured. After analyzing the results of the temperature and spectrum tests, the maximum temperature and the maximum frequency and maximum amplitude of the spectrum should be recorded.

### 2.2 Step 2: Soil Sample Extraction

Dry soil samples should be extracted from below the surface at the site in which the compressor outlet is located to ensure that the simulated soil is consistent with the soil present at the location in which the equipment is in use. The volume of soil extracted in this step should be is larger than the capacity of the test piece used in Step 4 to ensure that the soil sample can completely cover the test pieces.

### 2.3 Step 3: Sample Preparation

The samples are rectangular metal sheets; the length and width of the samples should be set in accordance with the relevant standards. The number of samples required is twice the number of test items in the standards; this ensures that the requirements for the performance comparison tests can be met. The material of the sample should be the same as, or similar to, the material from which the outlet pipe of the compressor is constructed. This ensures the simulation environment is as realistic as possible. The anticorrosion layer should be coated or winded on the sample; the coating or winding process should be the same as that used in the construction of the anticorrosion layer used on the actual equipment, and the anticorrosion material should be the same as that used on site.

### 2.4 Step 4: Experiment Preparation

**Figure 2:**
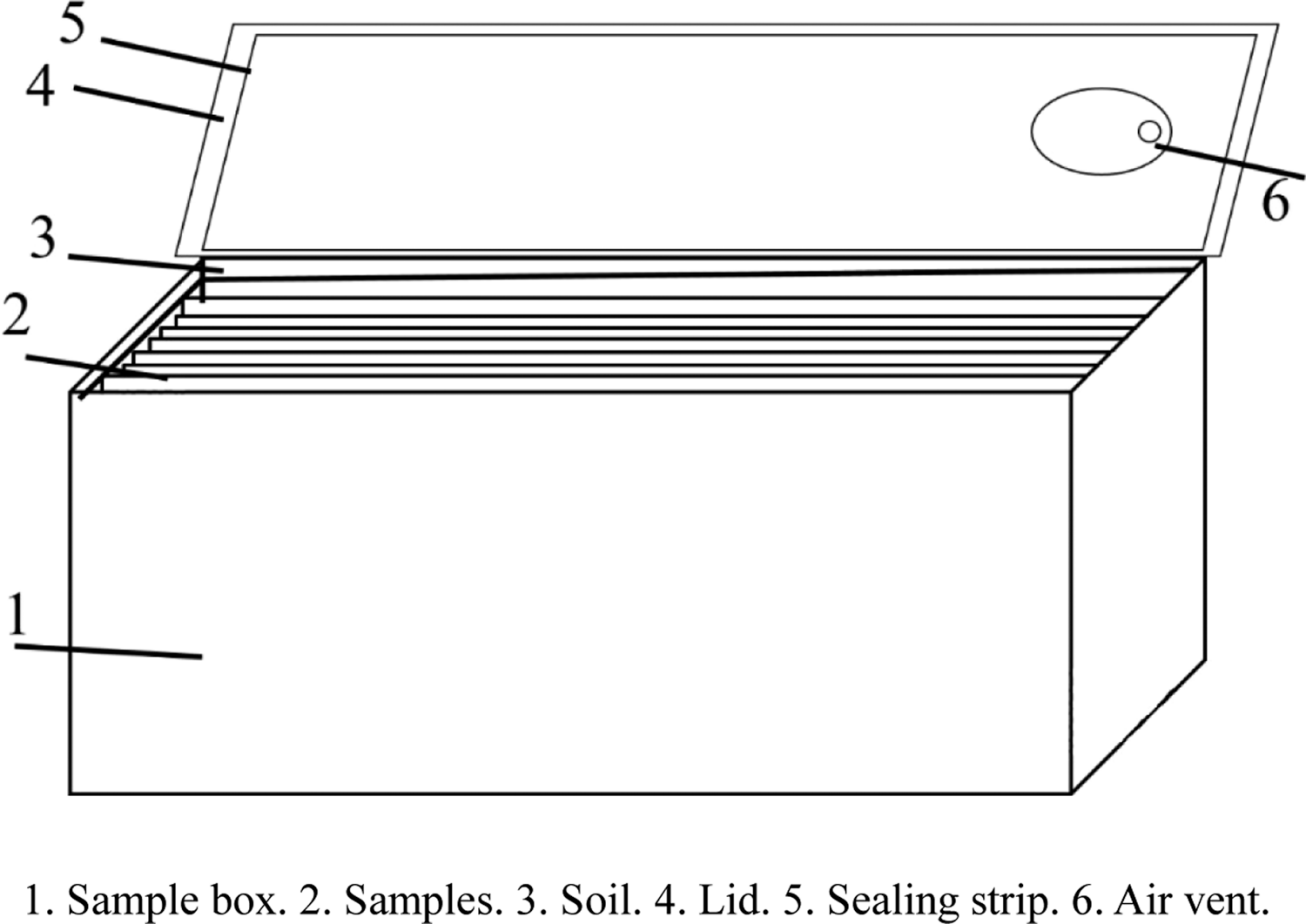
Schematic of the sample box and sample placement.

To prepare for the experiment, a sealed sample box should be used to replicate the service environment present in the on-site pipeline. A container with high-temperature resistance and air permeability that can be sealed should be used as the sample box (see Figure 2). The size of the sample box should be consistent with that of the test pieces, and the sample box should be put in the coupled temperature–vibration test box. Half of the samples should be placed into the sample box; the sample box should then be filled with the extracted soil (see Step 2). The sample box should then be filled with tap water to ensure the soil is saturated with water. This process simulates the harsh environment present in pipelines that are soaked in rainwater. The sample box should then be sealed with an air vent open. This slows the evaporation of water from within the sample box while ensuring that the water vapor in the sample box does not lead to the box being opened.

### 2.5 Step 5: Accelerated Testing

The sample box prepared in Step 4 is placed in the testing equipment that simultaneously exposes the samples to high temperatures and vibrations, and the temperature of the system is set to be the highest temperature observed in the measurements of the compressor outlet pipe in use. The vibration frequency and amplitude are set to be as the primary frequency and maximum amplitude observed in the vibration testing undertaken in Step 1. The experiment is set to be 30 days in duration.

### 2.6 Step 6: Performance Testing

At the end of the testing involving simultaneous exposure to high temperatures and vibrations, the service reliability of the anticorrosion coatings can be evaluated. The same evaluation can be undertaken on the conventional samples that were not subject to the simultaneous high temperature and vibration exposure for comparison; the results of these tests provide a basis for the selection of the anticorrosion coating for compressor outlet pipelines. The most common anticorrosion layers used in compressor outlet pipelines are three-layer polyethylene (3PE) anticorrosion coatings, fusion-bonded epoxy anticorrosion (FBE) coatings, and viscoelastic body anticorrosion coatings.

For the 3PE anticorrosion layers, in accordance with the standard GB/T 23257-2017,^3^ the tests focus on the items listed in Table 1.

**Table 1:** Type of Test to Be Used on the 3PE Anticorrosion Coatings According to Standard GB/T 23257-2017

item	test method	number of tests for each working condition	test conditions
stripping 65 °C, 48 h (mm)	Appendix D	3	coupled temperature–vibration testing, conventional testing
cathodic disbonding 70 °C, 28 days (mm)	Appendix D	3	
impact resistance (*J*)	Appendix L	3	
resistance to bending, 2.5°	Appendix E	3	

For the solvent-free epoxy coating, in accordance with the standard SYT 0315-2013,^5^ the tests focused on the items listed in Table 2.

**Table 2:** Type of Test to Be Used on the Solvent-Free Epoxy Coating in Accordance with Standard SYT 0315-2013

item	test method	number of tests for each working condition	test conditions
cathodic disbonding 65 °C, 48 h (mm)	Appendix C	3	coupled temperature–vibration testing, conventional testing
cathodic disbonding 65 °C, 28 days (mm)	Appendix C	3	
resistance to bending at –20 °C and 2.5°	Appendix D	3	
impact resistance (*J*)	Appendix E	3	
wear resistance (L/μm)	Appendix H	3	
adhesion (grade)	Appendix G		

In the case of the anticorrosion viscoelastic adhesive tape, in accordance with the standard SYT 5918-2011,^4^ the testing focuses on the items in Table 3.

**Table 3:** Type of Test to Be Used on the Viscoelastic Body Anticorrosion Testing, in Accordance with Standard SYT 5918-2011

item		test method	number of tests for each working condition	test conditions
thickness (mm)			4	coupled temperature–vibration testing, conventional testing
cathodic disbonding 65 °C, 48 h (mm)		GB/T 23257-2009 Appendix D	3	
cathodic disbonding 65 °C, 30 days (mm)		GB/T 23257-2009 Appendix D	3	
chemical immersion resistance, 7 days	10% H_2_SO_4_	SYT 0315-2005 Appendix I	3	
	10% HCl		3	
	5% NaOH		3	
	10% NaCl		3	

### 2.7 Step 7: Data Processing and Analysis

After the performance testing was completed, whether the performance established in the conventional anticorrosion layer testing and that established in the testing in which the samples were simultaneously exposed to high temperatures and vibrations met the standard requirements can be evaluated. If one of them does not meet the standard requirements, the anticorrosion layer can be considered to have failed to meet the service reliability level necessary for use in compressor outlet pipelines; if both meet the standard requirements, the anticorrosion layer can be considered to have met the service reliability requirements for use in compressor outlet pipelines.

## 3 Specimen Design

FBE powder coating and FBE–polypropylene tape anticorrosion coatings are the most common anticorrosion coatings used in early station pipelines, and their anticorrosion effects depend on the adhesion of the FBE to the base material. Thus, we consider only the FBE in this research.

**Figure 3:**
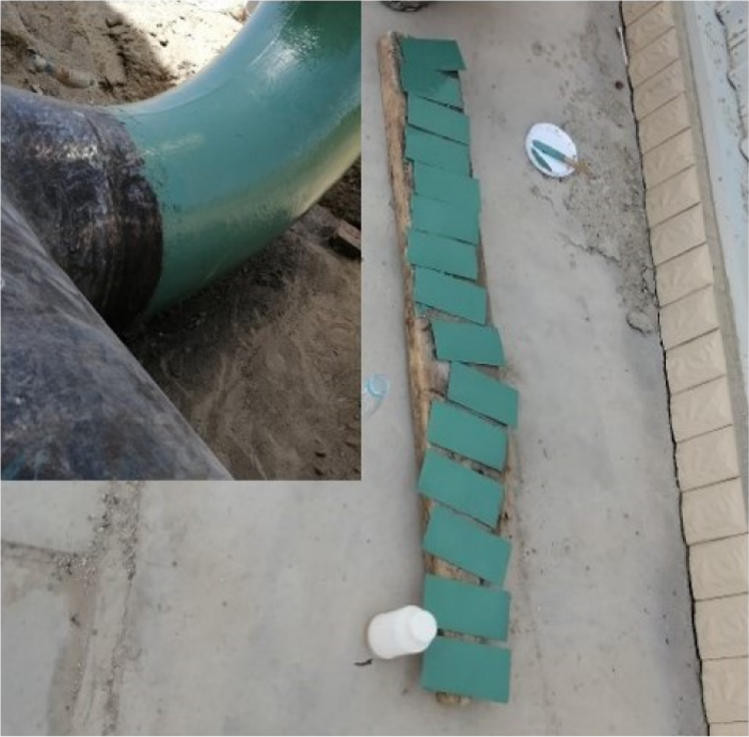
Preparation of the anticorrosive layer test specimens.

The anticorrosive layer test specimens used in this study were prepared according to the practices used in real applications. Prefabricated steel sheets were coated with the FBE coatings using the same process and products as used in standard use to ensure that the anticorrosion layer is consistent with that observed in real applications (Figure 3).

## 4 Experimental Process

### 4.1 Coupled High-Temperature and Vibration Exposure Experiment

The coupled high-temperature and vibration exposure test system, which was developed by our institute, was used to perform the coupled high-temperature and vibration testing of anticorrosion layers in a reduced timeframe; this test system simulates the working conditions of anticorrosion layers in a compressor outlet.

To meet the test requirement that the vibration and high temperature act simultaneously on the coating, the coupled temperature and vibration exposure equipment was developed in this research. This equipment is composed of a constant-temperature heating box, electric vibration table, linkage system, and a system that constantly monitors the state of the system. The equipment ensures that the anticorrosion layer test sample maintains a constant temperature, and the sample is simultaneously subject to vibration due to the action of the vibration table; thus, the temperature and vibration are coupled in this test system.

**Figure 4:**
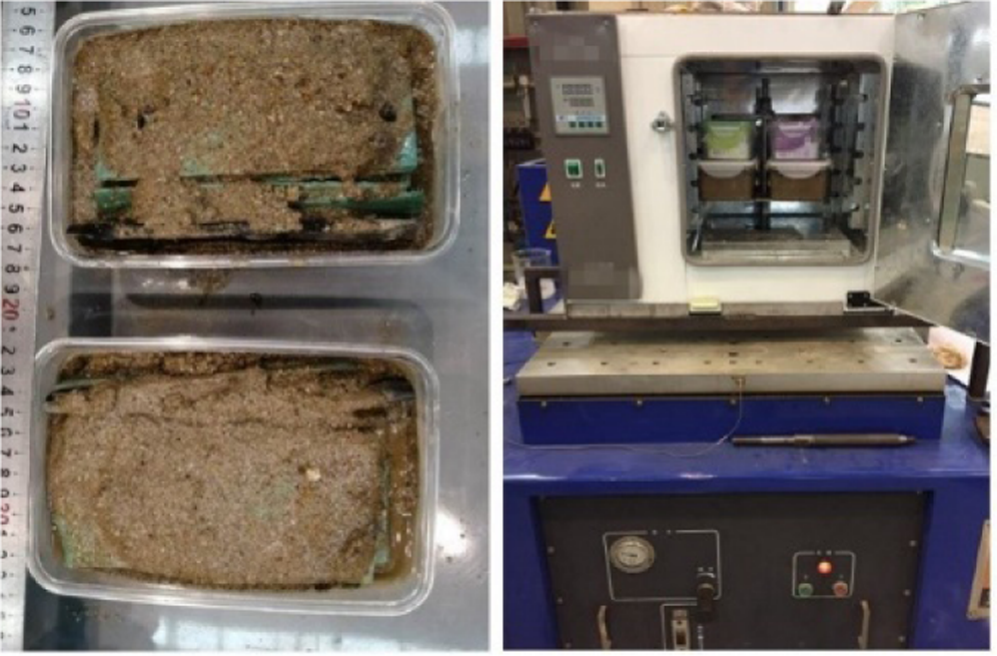
Experimental setup for high-temperature and vibration exposure.

The vibration loading used here has a primary frequency (500 Hz) and acceleration (80 m/s^2^) that were selected in accordance with the field tests, and the maximum temperature of the compressor outlet pipe was set to 70 °C.^1,2^ The specimens were buried in the sealed crisper containing the soil and water from the site and fixed to the vibrator using the metal splints; this setup ensures that the loading is in resonance with the vibrator while the samples are subject to heating, as shown in Figure 4. After 30 days of continuous and uninterrupted exposure to heat and vibration, the specimens were taken out for testing and analysis.

### 4.2 Comparison of the Properties of the Anticorrosive Layers

After the coupled temperature and vibration testing, the same tests were carried out on specimens acted on by the same vibration and at the same temperature but without the coupling of the temperature and vibration; this allowed us to establish an accurate comparison of specimens subject to different conditions and thus further insights into the degradation of the adhesion of the FBE anticorrosion layer.

In accordance with the standard SY/T 0315-2013 “Technological specification of external fusion-bonded epoxy coating for steel pipeline”,^6^ the FBE specimens subject to conventional and coupled temperature–vibration testing were subjected to thickness tests, bending tests, impact tests, abrasion tests, and adhesion tests.

Bending tests were carried out using a bending machine and a freezer at −20 °C, and the bending angle is 2.5°. Impact tests were carried out using an impact machine. The impact energy used in the testing undertaken here was 10 J. The pull-out adhesion tester was used to conduct a quantitative adhesive force test on the specimens. Prior to the adhesion tests, the specimens were kept in an incubator at a set temperature for 24 h.

## 5 Experimental Results and Discussion

### 5.1 Coating Thickness

The results of the thickness measurements are shown in Table 4. It can be seen that the coating thickness is not uniform, and the difference between the thickest point and the thinnest point is nearly 200 μm. The variation in thickness is large because the site construction environment has great influence on the coating quality. The coating thickness was found to be reduced after the simultaneous exposure to high temperatures and vibrations, but it is difficult to put this result in context as the coating was not initially uniform.

**Table 4:** Results of Thickness Testing on the FBE Coating

	thickness (μm)					
	before exposure to set temperature and vibration loading			after exposure to set temperature and vibration loading		
specimen number	point 1	point 2	point 3	point 1	point 2	point 3
a	928	871	521	604	723	587
b	934	745	634	765	579	876
c	698	523	579	764	685	587
average	714.78			685.56		
standard deviation	155.78			98.64		

### 5.2 Bending Tests at −20 and 2.5°

**Figure 5:**
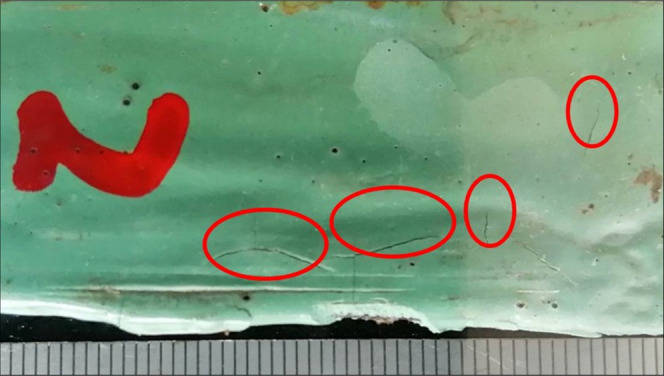
Surface cracks in the bending specimens.

The evolution of the coating surface when subject to the bending was observed, and the results are shown in Figure 5 and Table 5. It can be seen that the resistance to bending of the coating is reduced after simultaneous exposure to high temperatures and vibrations.

**Table 5:** Results of Bending Test on FBE Coatings

	conventional specimens			specimens after coupled temperature–vibration testing		
specimen number	a	b	c	1	2	3
cracks present	no	no	no	yes	yes	yes

### 5.3 Impact Resistance

**Figure 6:**
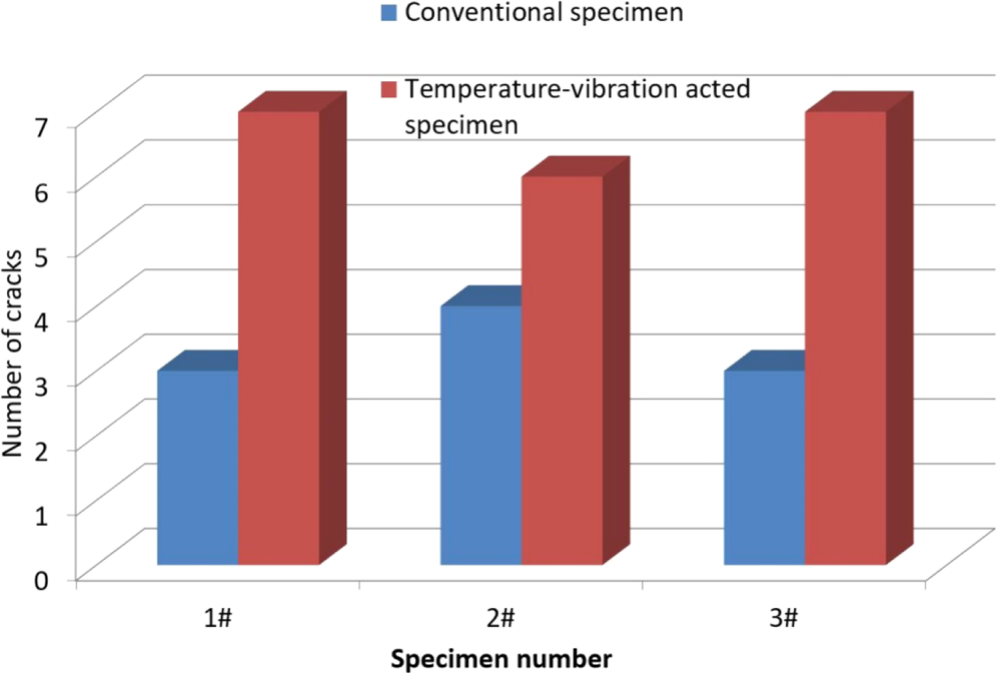
Number of cracks in the FBE coating specimens subject to impact testing.

The number of surface cracks in each of the samples subject to impact testing is shown in Figure 6. It can be seen that cracks appear at the impact points in each specimen, regardless of whether the specimen was subject to the conventional or coupled temperature–vibration testing. Thus, the impact resistance of the FBE coating can be seen to be poor. An electric spark leak detector was used for the detection of cracks. The detection voltage used here was 10 kV. Leakage points were observed at each of the three impact points in every specimen.

### 5.4 Abrasion Resistance

The results of the abrasion resistance tests are shown in Table 6. It can be seen that the abrasion resistance of the FBE coating applied using the standard process does not meet the requirements of the standard SY/T 0315-2013 at room temperature, and the abrasion resistance of the coating decreased significantly (by a factor of approximately 2/3) as a result of the coupled action of temperature and vibration.

**Table 6:** Results of the Abrasion Tests on the FBE Coatings

specimen number		δ1 (μm)	δ2 (μm)	abrasion resistance (L/μm)	average abrasion resistance (L/μm)
conventional specimens	a	471	419	1.3	1.3
	b	483	429	1.3	
specimen subject to simultaneous action of temperature and vibration	1	769	696	0.53	0.48
	2	842	751	0.43	

### 5.5 Adhesion

An adhesion tester was used to conduct an adhesion test on the specimen. To study the influence of exposure to high temperatures and vibrations on the adhesion of the coating, two tests were carried out. In test condition 1, the effect of exposure to high temperature was established. Prior to the tests, the specimens were placed in an incubator at a given temperature (40, 50, 60, and 70 °C) for 24 h. In test condition 2, the effect of exposure to high temperatures and vibrations was established. The specimens were subjected to the temperature of 70 °C (the highest temperature measured on the compressor outlet) and the maximum amplitude and frequency observed in the outlet pipeline for 30 days.

**Figure 7:**
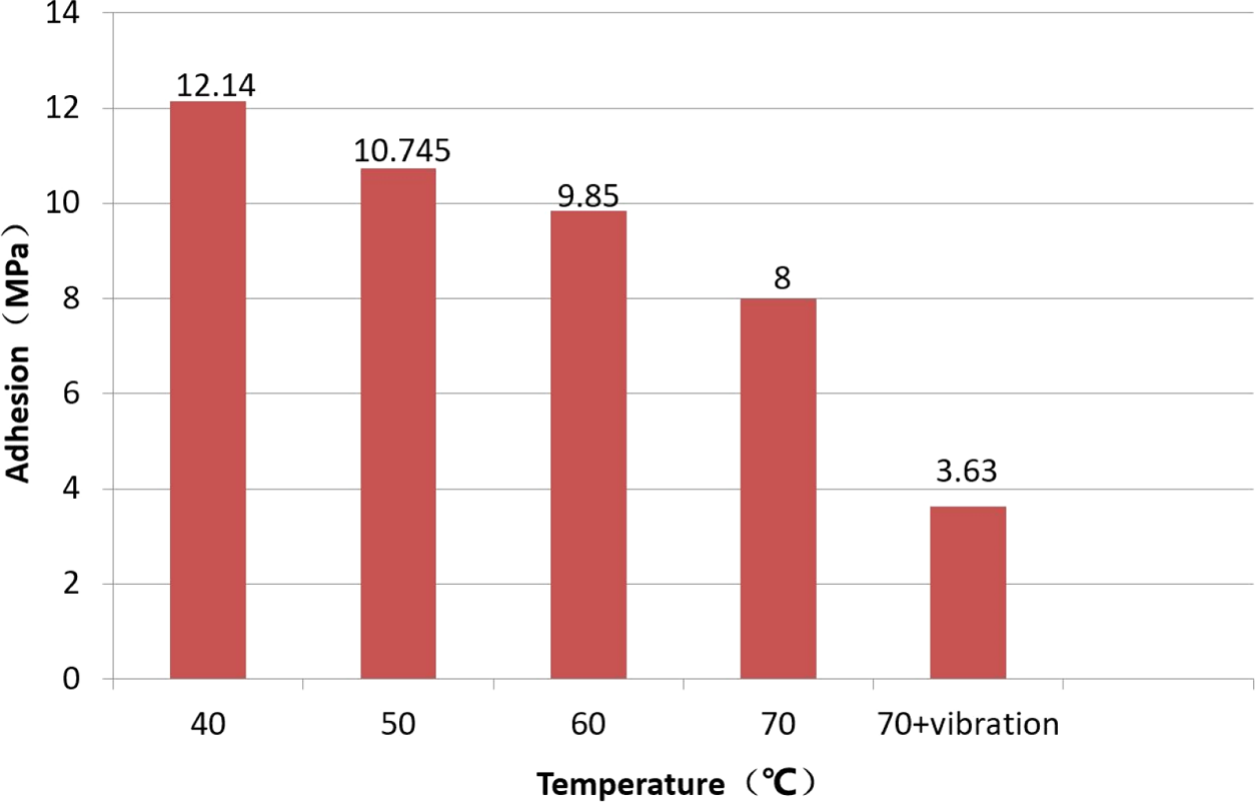
Adhesion of FBE coating as a function of temperature to which the sample was exposed.

The results of the adhesion tests are shown in Figure 7. As can be seen from the figure, the adhesion of the coating to the base material decreases with increasing temperature, and the adhesion of the coating after simultaneous exposure to vibrations and high temperatures decreases by a further 50% compared with the specimen exposed to the same temperature without vibrations.

The impact resistance of the FBE coating was found to fail to meet the standard requirements. With the increase in temperature to which the specimen was exposed, as well as the simultaneous exposure to high temperatures and vibrational loading, the impact resistance, bending resistance, abrasion resistance, and adhesion of the coating were seen to decrease significantly.

## 6 Discussion

### 6.1 Effect of Exposure to High Temperatures and Vibrations

**Figure 8:**
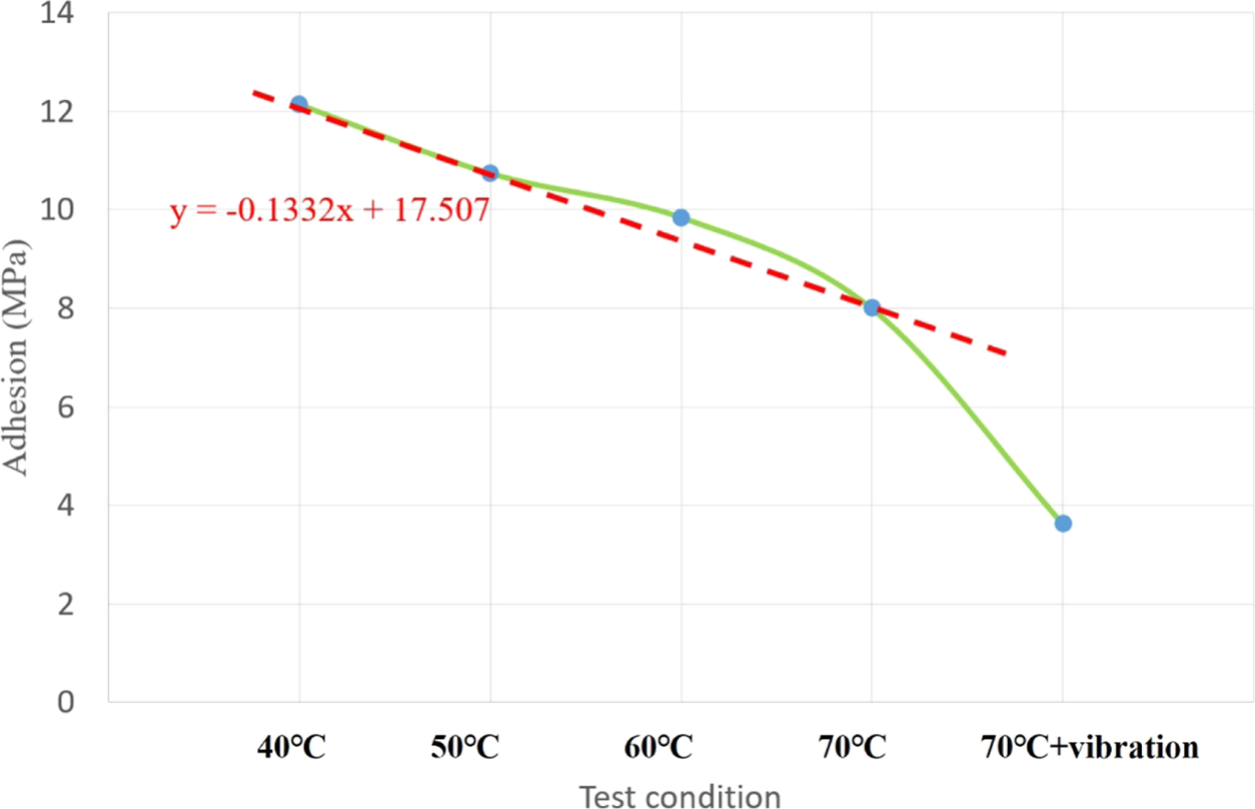
Adhesion of the anticorrosive layer subject to various experimental conditions.

A curve describing the adhesion of the anticorrosive layer to the base material when the specimen was subject to different conditions was established (see Figure 8). It was found that the adhesion of the anticorrosive layer decreased by approximately 1.3 MPa when the temperature was increased by 10 °C. When the specimen was also subject to vibrations, the adhesion of anticorrosive layer decreases to approximately half the value observed when the sample was exposed to the same temperature without exposure to vibrations. It can be seen that the temperature has a significant effect on the adhesion of the anticorrosive layer, but the simultaneous action of exposure to high temperatures and vibrations has a far larger effect on the adhesion of anticorrosive layer.

### 6.2 Microscopic Failure Mechanism

In this paper, moisture and soil, together with high temperatures and vibrations, were used to simulate, in a reduced time, a typical environment in which an anticorrosion layer is likely to be used. Here, we analyze the mechanism through which these effects are likely to act on girth welds from a microscopic perspective.

Water, oxygen, and ions are three elements that commonly cause corrosion. Anticorrosive coatings are a kind of high polymer film, which can inhibit the transmission of the aforementioned substances to differing degrees; thus, such a coating can reduce corrosion. When the temperature rises, from a microscopic point of view, the movement of water molecules is accelerated, and thus the permeability of the coating is increased. To reduce the water permeability, oxygen permeability, ion permeability, and water absorption of an anticorrosive coating, the cross-linking density and glass-transition temperature, *T*_g_, of the coating should be increased; this makes the epoxy coating more dense and increases its shielding performance.

The failure mechanism of anticorrosive coatings primarily depends on the location of the pipeline, its construction quality, and the operation conditions; water absorption of the coating is also a factor in the failure of a coating.

**Figure 9:**
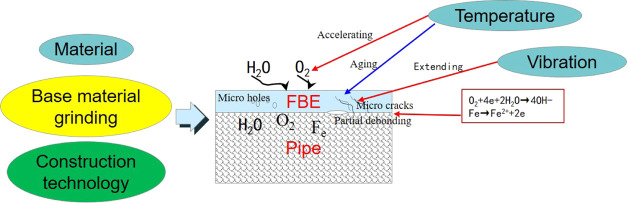
Schematic of the damage mechanism of FBE coatings when exposed to high temperatures and vibrations.

The damage mechanism of the anticorrosion layer of a buried pipeline at a compressor outlet is shown in Figure 9. Due to the nature of the raw materials, surface treatments, coating processes, the FBE coating will exhibit pores, cracks, local debonding, and other defects. Under the coupled effect of exposure to high temperatures and vibrations, as well as the presence of a corrosive environment (H_2_O, O_2_), any initial defects will gradually increase in size, resulting in a gradual decrease in the adhesion of the coating to the base material. In buried pipelines, the pipeline is not completely surrounded by water after rain, and a certain concentration of oxygen will be present in surrounding the pipeline, which will lead to aging and cracking of the anticorrosion layer; eventually, in such conditions, the pipeline will be subject to significant corrosion. This is because oxygen captures electrons, and, in the presence of water, this generates OH^–^ ions, resulting in pipeline corrosion. This is described by
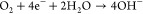


Oxygen obtains electrons from water molecules to form OH^–^ ions.
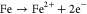


The metal loses electrons and combines with hydroxide ions to form hydroxides, which corrodes the surface of the pipe, and the corrosion products further lead to the debonding of the coating.

**Figure 10:**
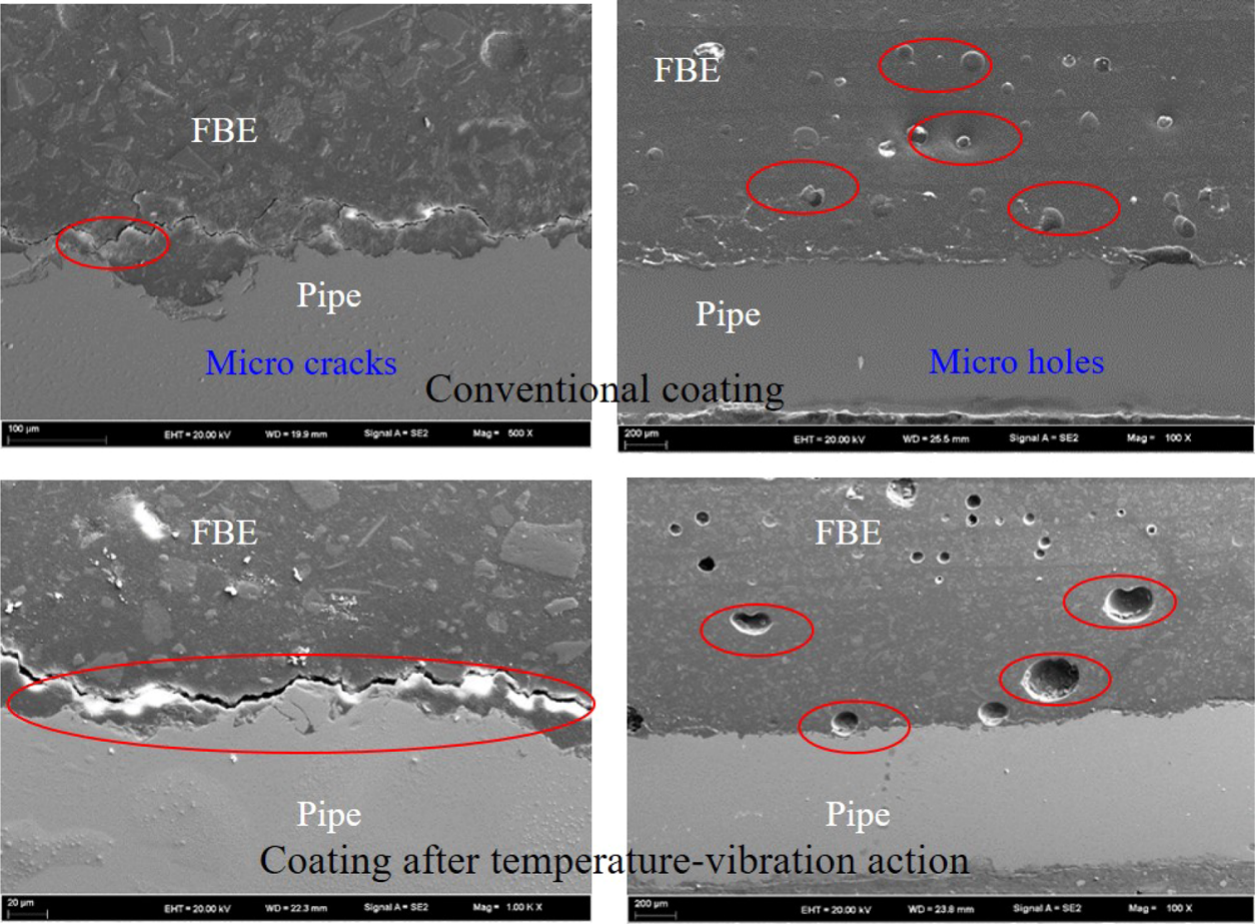
SEM observation of the anticorrosion coatings.

Observations on the microscopic scale were made of the conventional coating sample and the sample after exposure to both high temperatures and vibrations. Scanning electron microscopy (SEM) was used to observe the microstructure of the samples. It was found that holes, cracks, and local debonding defects could be observed in the coatings subject to conventional testing; in the samples subject to the simultaneous exposure to high temperature and vibrations, the holes, cracks, and debonding defects were larger than those observed in the samples subject to conventional testing, as shown in Figure 10. The experimental results demonstrate that the combined effect of exposure to high temperatures and vibrations accelerates the expansion of initial defects within the coatings, which leads to the eventual failure of the coating.

To analyze the evolution of the microstructure of the anticorrosive layer as a result of the exposure to high temperatures and vibrations, the size distribution of the holes in a given area of two anticorrosion coating samples (one not subject to simultaneous exposure to high temperatures and vibrations and the other subject to high temperature and vibrations) was calculated. The number of holes in the anticorrosive layer per square meter was estimated. The results are shown in Table 7. It can be seen that in the ordinary sample, the number of holes per unit area is large, but the hole diameter is small; in the sample exposed to high temperatures and vibrations, the number of holes per unit area is relatively small, but the hole diameter is larger than that observed in the ordinary sample. Due to the coupled effect of exposure to high temperatures and vibrations, the original pores in the anticorrosive layer expand and merge, developing into large pores.

**Table 7:** Number of Microholes Per Square Meter of Sample

	number per square meter	
diameter of microhole (μm)	conventional coating sample	sample exposed to high temperatures and vibrations
20	5840	2596
40	4180	3526
60	2016	2561
>60	1460	3569
combined	13 496	12 252

## 7 Conclusions

In this article, specimens of FBE anticorrosive coatings were tested, experimental equipment suitable for investigating the coupled action of exposure to high temperatures and vibrations was developed, and the soil samples obtained from actual compressor outlet pipeline locations were used to conduct the tests on compressor outlet anticorrosion coatings in a reduced time frame. Through the analysis of various performance indicators of anticorrosive coatings, the following conclusions can be drawn: the FBE anticorrosion layers do not meet the requirements related to coupled high temperature and vibration exposure, anti-impact performance, abrasion resistance, and bending resistance. It is thus suggested that FBE anticorrosion layers should be used on buried compressor outlet pipelines with extreme caution.

The failure mechanism of FBE coatings subject to simultaneous exposure to high temperatures and vibrations was established. FBE coatings exhibit initial pores, microcracks, local debonding, and other defects as a result of the manufacturing techniques used. Under the coupled effect of exposure to high temperatures and vibrations, the original defects gradually increase in size, which leads to a decrease in the adhesion of the coating to the base material.
